# Analysis of Perception Accuracy of Roadside Millimeter-Wave Radar for Traffic Risk Assessment and Early Warning Systems

**DOI:** 10.3390/ijerph20010879

**Published:** 2023-01-03

**Authors:** Cong Zhao, Delong Ding, Zhouyang Du, Yupeng Shi, Guimin Su, Shanchuan Yu

**Affiliations:** 1 Key Laboratory of Road and Traffic Engineering of the Ministry of Education, Tongji University, Shanghai 201804, China; 2 Shanghai Pudong Development (Group) Co., Ltd., Shanghai 201204, China; 3 Shanghai SH Intelligent Automotive Technology Co., Ltd., Shanghai 201805, China; 4 Shanghai SEARI Intelligent System Co., Ltd., Shanghai 200063, China; 5 National Engineering and Research Center for Mountainous Highways, China Merchants Chongqing Communications Research & Design Institute Co., Ltd., Chongqing 400067, China

**Keywords:** intelligent transportation systems, traffic safety, risk warning, roadside perception, millimeter-wave radar

## Abstract

Millimeter-wave (MMW) radar is essential in roadside traffic perception scenarios and traffic safety control. For traffic risk assessment and early warning systems, MMW radar provides real-time position and velocity measurements as a crucial source of dynamic risk information. However, due to MMW radar’s measuring principle and hardware limitations, vehicle positioning errors are unavoidable, potentially causing misperception of the vehicle motion and interaction behavior. This paper analyzes the factors influencing the MMW radar positioning accuracy that are of major concern in the application of transportation systems. An analysis of the radar measuring principle and the distributions of the radar point cloud on the vehicle body under different scenarios are provided to determine the causes of the positioning error. Qualitative analyses of the radar positioning accuracy regarding radar installation height, radar sampling frequency, vehicle location, posture, and size are performed. The analyses are verified through simulated experiments. Based on the results, a general guideline for radar data processing in traffic risk assessment and early warning systems is proposed.

## 1. Introduction

With the rapid development of cooperative vehicle-infrastructure systems (CVISs), roadside perception systems play an essential role in modern transportation systems because they can provide a panoramic view of the road traffic and address inadequate vehicle detection [1]. Roadside sensors, such as millimeter-wave (MMW) radars, cameras, and lidars, can track multiple vehicles and perceive real-time traffic information, which can support cooperative vehicle-infrastructure applications such as real-time traffic monitoring and management [2,3], path planning [4], and speed control [5,6]. Compared with cameras and lidars, millimeter-wave radars have advantages such as good environmental adaptability, long detection ranges, and accurate velocity measurement [7], making them the most widely used roadside sensors in roadside perception systems. Using short-wavelength electromagnetic waves, MMW radar emits signals and captures those reflected by objects in vehicles’ paths to determine the real-time position and velocity of vehicles on the roads.

**Figure 1:**
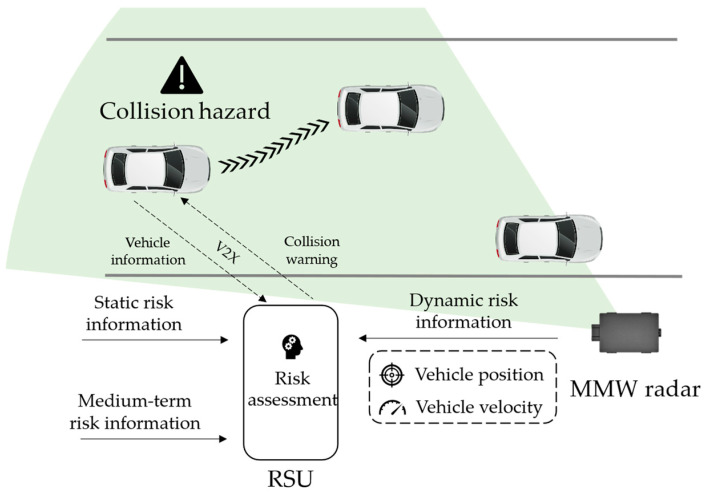
A typical roadside MMW radar-based traffic risk assessment and early warning system.

For roadside MMW radar-based traffic risk assessment and early warning systems (Figure 1), MMW radar can provide real-time position and velocity measurements as a source of dynamic risk information. Consequently, a risk assessment algorithm integrates such information with static risk information (high precision road maps) and medium-term risk information (weather, pavement distress, etc.) to predict potential hazards, such as collision. When a connected vehicle enters the communication range, it provides its location and identity information to a roadside unit (RSU) through a Vehicle-to-Everything (V2X) network. The RSU performs vehicle matching and sends back the warning message, if it exists. 

However, due to the measuring defects of MMW radars, challenges such as low angular resolution and poor elevation measurement have limited their measurement accuracy, leading to data quality issues such as vehicle positioning errors [8,9]. Positioning errors regarding vehicles on the roads may lead to the misperception of the vehicle motion and interaction behavior, potentially causing inaccurate decision making or fatal crashes in transportation systems [10]. Therefore, it is necessary to investigate the factors influencing MMW radar positioning accuracy to provide an insight into sensor development [11] and data processing [12]. In this paper, we analyze the factors influencing the positioning accuracy of roadside traffic MMW radar. We perform a qualitative analysis of the radar positioning error regarding radar installation factors and vehicle target characteristics, such as vehicle location, posture, and size. Subsequently, we verify the analysis through simulated experiments. Based on the results, we propose a guideline for MMW radar data processing in traffic risk assessment and early warning systems. 

The remainder of the article is organized as follows: Section 2 reviews the related work about traffic risk assessment and early warning systems, and radar perception accuracy analysis. Section 3 analyzes the factors influencing the positioning accuracy based on the detection principles of MMW radar. Section 4 presents the simulation results from analyzing the influencing factors discussed in Section 3. Section 5 introduces a guideline for radar data processing in traffic risk assessment and early warning systems. Section 6 summarizes the conclusions and limitations of the study, and future work. 

## 2. Related work

### 2.1. Traffic Risk Assessment and Early Warning Systems

The task of traffic risk assessment and early warning systems is first to identify the high-risk traffic environment or behavior and then send the warning message to the relevant parties. For microscopic level risk assessment, multiple indicators have been proposed to quantify the traffic risk. The longitudinal risk indicators include time to collision (TTC), inverse time to collision (TTC-I), time headway (THW), deceleration rate to avoid crash (DRAC), etc. [13,14,15]. Lateral risk indicators include time-to-lane crossing (TLC), variable rumble strip (VRBS), etc. [16,17]. To quantify the traffic risk in a spatial continuous manner, the use of an artificial field has also been proposed. In [18], authors use safety field theory to estimate crash risk and severity by modeling the safety-aware interactions of various road users.

Whether it is for risk indicators or artificial field calculation, real-time risk assessment relies heavily on vehicle location and speed detections. The positioning error may cause misperception of the vehicle motion and interaction behavior, causing false alarms and warning failure. 

### 2.2. Radar Perception Accuracy Analysis

Most work on radar measurement accuracy analysis focuses on analyzing the signal processing procedure. The theoretical limits of frequency-modulated continuous wave (FMCW) ranging accuracy due to the tolerance of crystal oscillators were analyzed [19]. In [20], the impact of the frequency ramp nonlinearity, phase noise, and signal-to-noise ratio (SNR) on FMCW radar accuracy were mathematically analyzed and validated with real measurements. In [21], the authors compared the SNR characteristics of the angle estimation error using Capon [22] and MUSIC [23] beamforming algorithms. A phasor statistical analysis was applied in [24] to analyze the influence of Gaussian white noise, static, and adjacent clutter. Another line of work focused on the measurement error caused by the measurement environment. Atmospheric factors such as fog and rain have also been investigated. In [25], the attenuation and group delay effects on MMW propagation in clouds were theoretically analyzed and verified using artificial fog and a metal plate. In [26], the propagation of MMW in the atmosphere was modeled and validated with numerical experiments. The above-mentioned research used an ideal target (metal plate, metal sphere, or corner reflector) to verify the influence of signal processing methods and the measurement environment since they all considered the target as a single point in radar measurement. Few studies have focused on the error characteristics regarding the features of the detection target. 

However, for applying roadside perception in transportation systems, MMW radar locates vehicles based on the bounding box inferred from the radar point cloud rather than a single radar radiation point. Lack of sufficient data points and side-only detections [27] can cause erroneous estimation of the bounding box, leading to a larger positioning error than that caused by electromagnetic wave propagation characteristics. Therefore, the vehicle shape and posture and the point cloud distribution are also major factors contributing to the positioning accuracy, as well as the above conventional factors. To the best of our knowledge, no study has focused on the MMW radar positioning accuracy of vehicles on the roads. 

## 3. Factors Influencing Positioning Accuracy

### 3.1. Radar Detection Principles

**Figure 2:**
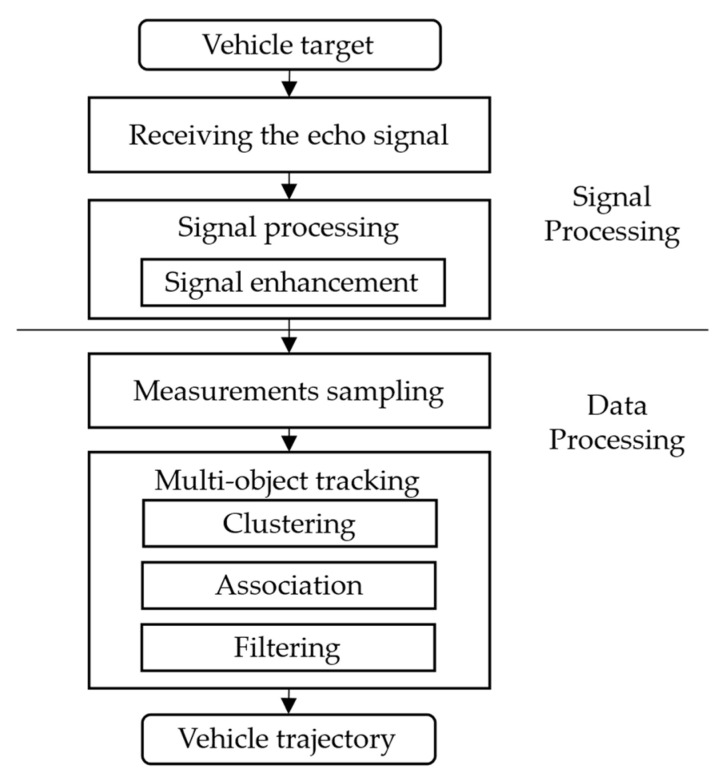
MMW radar data processing procedure.

Figure 2 shows a typical detection procedure for traffic MMW radar data to locate the vehicles on the roads. It involves two modules: a signal processing module and a data processing module. The signal processing module estimates the target range and angle by processing the echo signal; the positioning error can arise from limitations of the signal processing hardware and algorithms. The data processing module identifies and tracks the positions of the target vehicles from the point cloud data; uneven distribution of the measured point clouds can also cause positioning deviations on the target level. Specific principles of the signal and data processing and the errors thus generated are discussed as follows.

#### 3.1.1. Signal Processing Module

**Figure 3:**
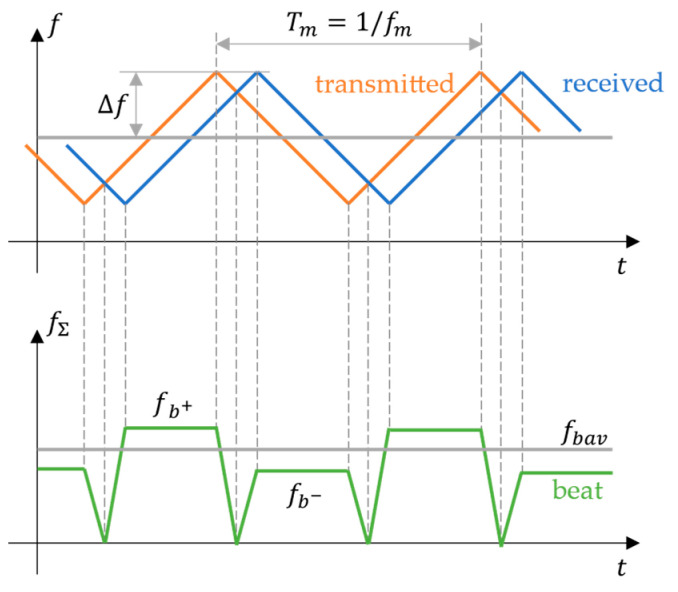
FMCW ranging: f shows the frequencies of the transmitted and received signals, $f_{\Sigma }$ the frequency of the beat signal, Δf the maximum frequency offset, $f_{b^{+}}$ and $f_{b^{-}}$ the positive and negative beat frequencies, respectively $f_{\mathrm{bav}}$ the average beat frequency, and $f_{m}$ and $T_{m}$ the frequency and period of the triangular wave, respectively.

MMW radar systems measure the object range and angle by extracting relevant information from echo signals. The signal processing module eliminates unwanted signals (such as clutter), processes or enhances the echo signal, then calculates the target range and angle. Typically, an FMCW ranging method [28] uses a signal whose frequency varies according to a periodic triangular wave, as shown in Figure 3. The target radial range R can be found with

(1) $$R=\frac{c}{8\Delta f}\frac{f_{b^{+}}{+f}_{b^{-}}}{{2\;f}_{m}},$$

where c denotes the speed of light, Δf the maximum frequency offset, $f_{b^{+}}$ and $f_{b^{-}}$ the positive and negative beat frequencies, respectively, and $f_{m}$ the frequency of the triangular wave. 

The ranging error ΔR is evaluated as

(2) $$\Delta R=\frac{c}{8\Delta f}\frac{{\Delta f}_{\mathrm{bav}}}{f_{m}},$$

where ${\Delta f}_{\mathrm{bav}}$ is the average beat frequency error and $\frac{{\Delta f}_{\mathrm{bav}}}{f_{m}}$ the mean average beat frequency error in the modulation period. It is shown that in FMCW ranging, the ranging error is independent of the ranging distance or the operating frequency but inversely proportional to the signal bandwidth Δf.

**Figure 4:**
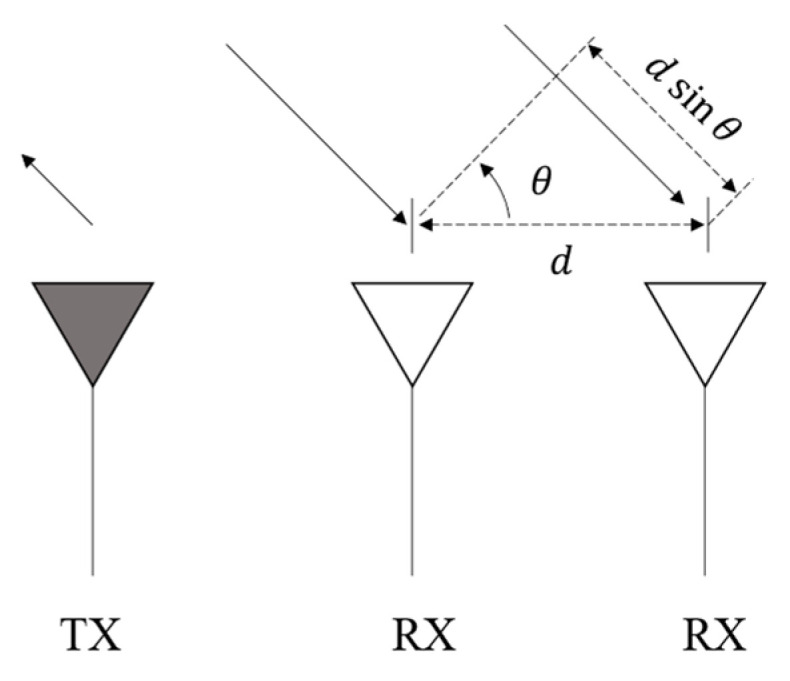
MMW radar angle measurement: TX and RX are the transmitting and receiving antennas, respectively, d the distance between two antennas, and θ the target angle.

Regarding the angular measurement, a phase-based method uses the phase difference between echo signals received by multiple antennas. As shown in Figure 4, the phase difference φ can be calculated with

(3) $$\varphi =\frac{2\pi }{\lambda }d\sin\theta ,$$

where λ is the radar wavelength, d the distance between two antennas, and θ the target angle. The target angle can then be determined by comparing the phases of the two echo signals with a phase meter

(4) $${\theta =\sin}^{-1}\frac{\varphi \lambda }{2\pi d}.$$

For the angular measurement, the measurement error can be derived as

(5) $$\Delta \theta =\frac{\lambda }{2\pi d\cos\theta }\Delta \varphi .$$

It is shown that the measuring accuracy can be improved by using a more accurate phase meter (decreasing Δφ) or increasing the distance between antennas (increasing d). Moreover, the angular measurement is more accurate near the normal direction of the antenna (higher cosθ).

#### 3.1.2. Data Processing Module

**Figure 5:**
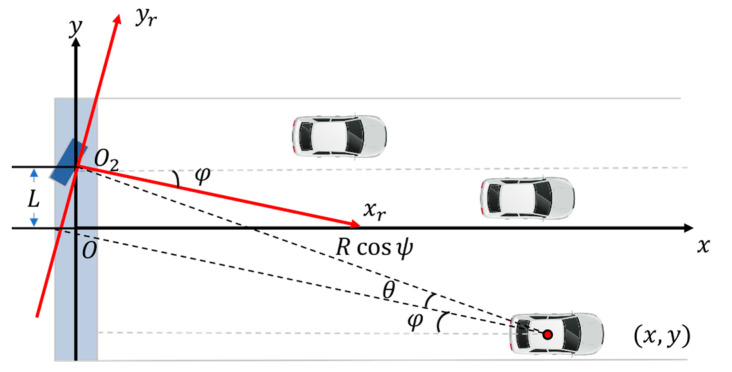
Detection coordinate transformation.

Once the target range and angle are acquired, the detection results must be transformed into a lane coordinate system for further application in transportation systems. As shown in Figure 5, the lane coordinate system is defined as xOy, with the driving direction defined as the y-direction. The radar module is installed over the center of the lane with deviation L in the x-direction and facing the driving direction with yaw angle φ and pitch angle ψ. The polar position measurement data collected by the radar module (R, θ) can be considered as a two-dimensional measurement in the radar coordinate system $x_{r}O_{2}y_{r}$, with the installation position as the coordinate origin $O_{2}$ and the direction of the radar central beam as the $y_{r}$-direction. The projection of the radar detection in the lane coordinate system (x, y) is then calculated as

(6) $$\{\begin{array}{c} x=R\cos\psi \sin(\varphi +\theta )-\;L\\ y=R\cos\psi \cos(\varphi +\theta )\;\;\;\;\;\; \end{array},$$

where radar installation parameters L, φ, and ψ are acquired through extrinsic calibration. 

In the process of coordinate transformation, the calibration error can also affect the position output, as shown in

(7) $$\{\begin{array}{l} \Delta x=-\;R\sin\psi \sin\left(\varphi +\theta \right)\Delta \psi +R\cos\psi \cos\left(\varphi +\theta \right)\Delta \varphi \;-\;\Delta L\\ \Delta y=-\;R\sin\psi \cos\left(\varphi +\theta \right)\Delta \psi \;-\;R\cos\psi \sin\left(\varphi +\theta \right)\Delta \varphi \; \end{array}.$$

When the target is further away (larger R), the installation posture estimation errors Δψ and Δφ can cause a larger positioning error, while the translation estimation error ΔL causes a systematic lateral positioning error.

Moreover, for the roadside perception scenario in transportation systems, MMW radar modules usually have sufficient resolution to obtain several measured radar points from the target vehicle, forming the point cloud data. In the data processing module, the detected points are sampled and collected by a multitarget tracking algorithm to generate the tracking results. 

**Figure 6:**
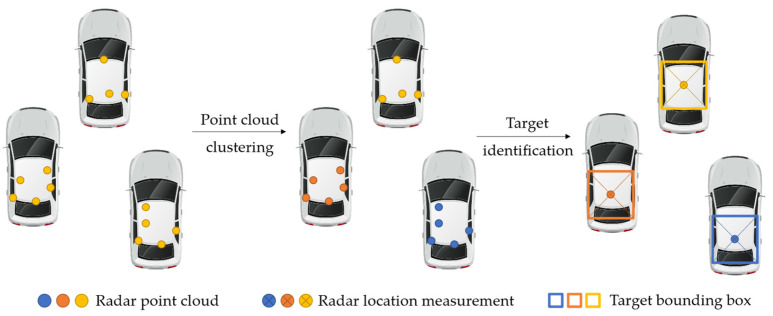
Radar point cloud data processing.

Specifically, as shown in Figure 6, a point cloud clustering algorithm divides the detected points into individual zones to identify different targets. Traditionally, there are grid-based clustering and density-based clustering. Grid-based clustering algorithms first spatially divide the measurements into multiple grids, and then perform clustering on the basis of the statistical value calculated at each grid. Typical algorithms include WaveCluster, STING, and CLIQUE [29]. The grid-based approach can achieve a better processing time, as the grid structure makes the original dense point cloud sparser. Density-based algorithms include DBSCAN, OPTICS, DENCLUE [30], etc. They detect clusters by finding the ‘density-reachable’ points, and thus are capable of producing clusters of arbitrary shapes [31]. 

When the classifier identifies the selected point cloud as a pre-learned category (truck, bus, or car), a bounding box is provided to estimate the area occupied by the target vehicle. Consequently, the anchor point for each bounding box is selected to form an association with historical trajectories. Finally, the position measurement is corrected by a filter algorithm to acquire the optimal position estimation. Probabilistic and hierarchical approaches are two of the main techniques used in data association [32]. Considering the filtering algorithm, the probabilistic methods can be distributed between the Kalman filter (KF) and the particle filter (PF). Popular techniques include the Probability Data Association Filter (PDAF) [33], Global Nearest Neighbor (GNN) [34], Probability Hypothesis Density (PHD) filter [35], etc. The hierarchical methods do not require the filter to provide state and covariance estimations as with KF or PF. Popular methods include Hungarian-algorithm-based methods, LSTM-based methods, tracklet association, etc. [32]. 

### 3.2. Analysis of the Factors Influencing Positioning Accuracy

In the roadside perception scenario for the MMW radars, a vehicle’s relative position and posture change continuously under different traffic conditions and driving behaviors. Moreover, due to insufficient measured points and side-only detections, the distribution of the radar point cloud for the vehicle also changes continuously with the vehicle’s shape and posture, causing positioning deviation at the target level. We propose to investigate this phenomenon from the perspective of the vehicle location, posture, and size so that insight can be provided for the higher-level application of radar data. 

#### 3.2.1. Radar Installation Height

**Figure 7:**
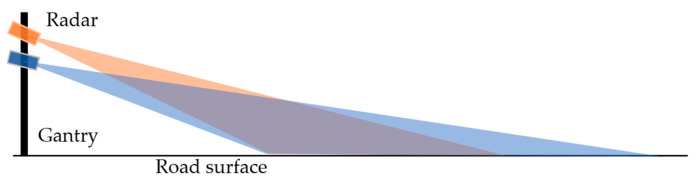
Radar FOV at different installation heights.

The installation location of the radar module affects the aspect of the view from which the vehicle target is observed. A higher installation position of the radar module can reduce the probability of target occlusion [11]. However, as a radar module usually has a limited elevation field of view (FOV) to monitor the same sections of road, the pitch angle of the radar module is larger when it is installed at a higher location, as shown in Figure 7, limiting its FOV on the road section. Regarding the positioning accuracy, when the radar module is installed at a higher position, due to the limited FOV, the error characteristics are scaled in the longitudinal direction, so that when the vehicle is driving away from the radar module, the longitudinal measurement from a radar installed at a higher position may deviate more from the ground truth, as the vehicle is closer to the edge of its FOV. This phenomenon is further explained by analyzing the influence of vehicle location. 

#### 3.2.2. Radar Sampling Frequency

**Figure 8:**
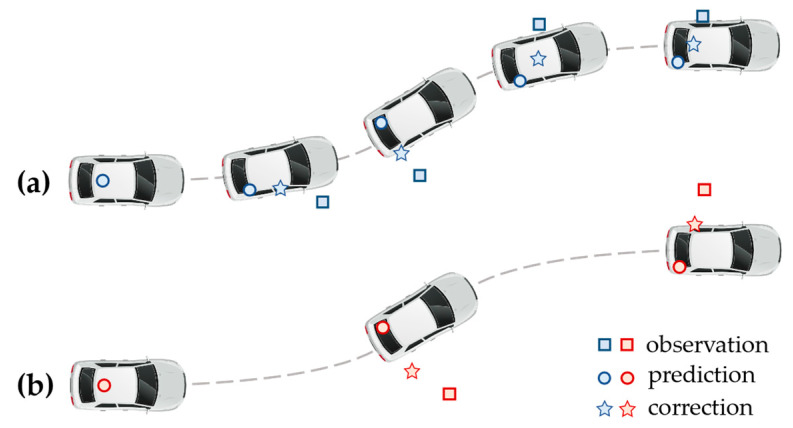
Variation in the tracking results regarding radar sampling frequency: (**a**) high sampling frequency; (**b**) low sampling frequency.

The sampling frequency can affect the positioning accuracy through the tracking algorithm. The tracking algorithm gives position estimation of the target vehicle (correction) by combining the observation and the prediction. As shown in Figure 8, a higher sampling frequency enables the tracking algorithm to predict the future position of the target vehicle more accurately. Therefore, despite the range and angular resolution of the MMW radar being the same, radar with a higher sampling frequency can produce more accurate vehicle trajectories. However, when the movement of the vehicle is easy to model (constant velocity, constant acceleration, etc.), radar with a higher sampling rate may have little advantage since the future position can be well predicted by the tracking algorithm. 

#### 3.2.3. Vehicle Location

**Figure 9:**
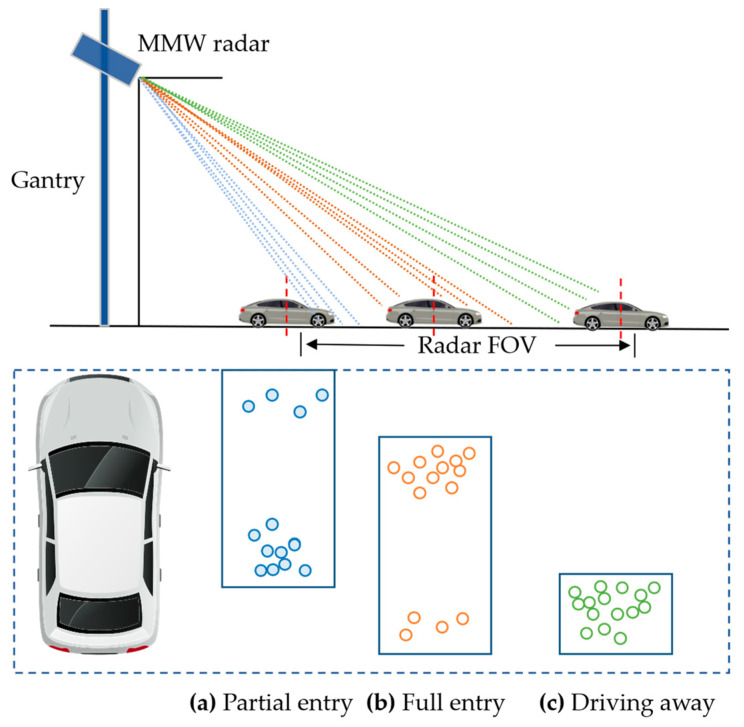
Variation in the effective scatterer regarding longitudinal vehicle location.

Radar detections of a target vehicle rely on the effective target scatterer, the part of the vehicle that can be illuminated by the radar beam. An MMW radar has a large monitoring range and is often installed at a certain pitch angle and far away from the target vehicle. Due to the influence of radar angle resolution and signal attenuation, as the vehicle passes through the FOV of the radar, the effective vehicle scatterer varies as shown in Figure 9. Table 1 summarizes the variation in the effective scatterer and radar measuring points when the vehicle is at different longitudinal locations. As the vehicle drives further away, the number of measured radar points decreases, the radar cross-section (RCS) decreases, and the effective scatterer moves closer to the rear of the vehicle. When the distance between the target and radar exceeds a certain range, the effective scatterer is limited to the rear of the vehicle, causing a large positioning error in the longitudinal direction. 

**Figure 10:**
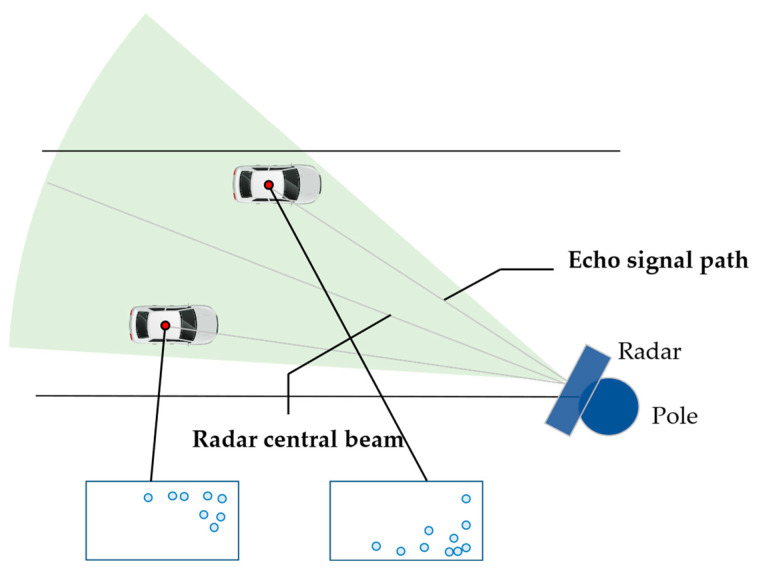
Variation in point cloud distribution regarding lateral vehicle location.

The lateral location of the vehicle can also affect the distribution of the radar point cloud on the vehicle body, as shown in Figure 10. When the vehicle is at the right side of the radar central beam, most of the detection points are distributed on the left part of the vehicle body, causing the radar location measurement to drift to the left and vice versa. 

**Figure 11:**
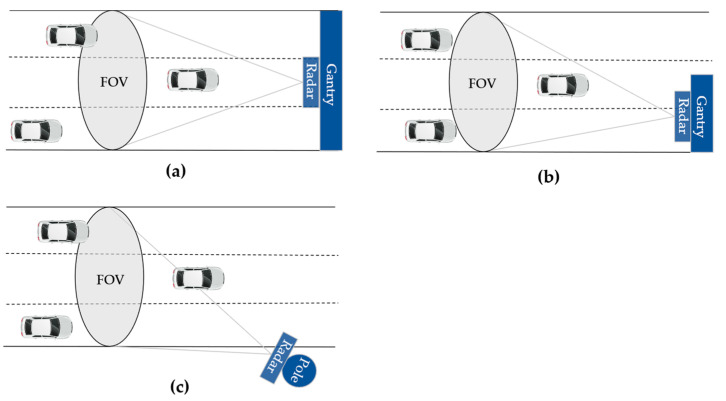
Radar installation schemes: (**a**) front top mounting; (**b**) side top mounting; (**c**) side mounting.

As shown in Figure 11, different radar installation schemes may similarly affect the vehicle positioning accuracy. When the radar is mounted on the center of the gantry, as shown in Figure 11a, the characteristics of the longitudinal and lateral radar measurements are as discussed previously. When the radar is installed on one side of the road, as shown in Figure 11b,c, the lateral positioning of the vehicles on the other side of the road may be subject to larger errors since the vehicle locations deviate more from the radar central beam. 

#### 3.2.4. Vehicle Posture

**Figure 12:**
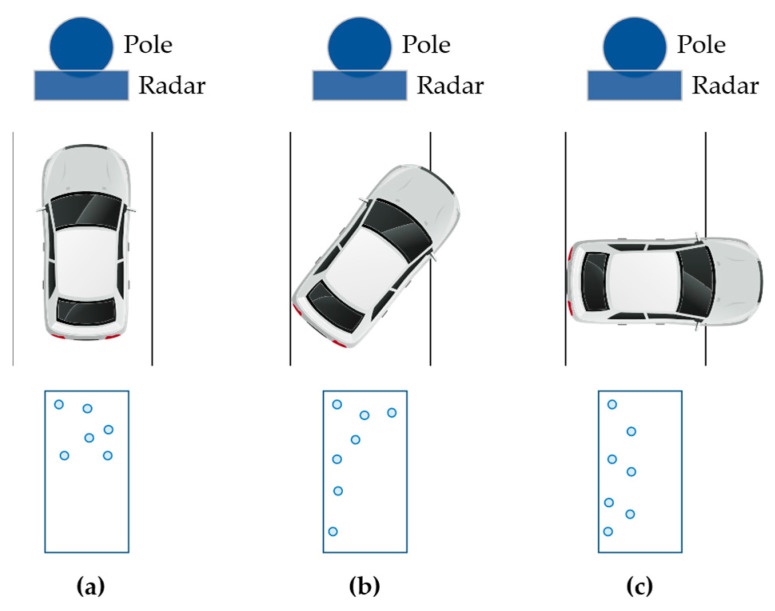
Variation in point cloud distribution regarding vehicle posture: (**a**) yaw angle 0°; (**b**) yaw angle 45°; (**c**) yaw angle 90°.

As the relative posture of the vehicle in relation to the radar module changes under different driving scenarios and radar installation conditions, the shape of the effective scatterer and distribution of the point cloud on the vehicle body change accordingly, as shown in Figure 12. When the vehicle faces the radar module, most of the measured points are scattered at the front. As the vehicle gradually turns to the right, the measured points gradually drift to the left side of the vehicle. When the side of the vehicle is facing the radar module, the longitudinal length of the vehicle in world coordinates drops drastically, leading to significantly different error characteristics. 

#### 3.2.5. Vehicle Size

Different types of vehicles have different RCS features. The total RCS of a target can be calculated as the vector sum of each scatterer (such as a sphere, cylinder, or plane), as shown in

(8) $$\sigma ={\left|\sum_{k}\sqrt{\sigma_{k}}e^{\frac{{j4\pi d}_{k}}{\lambda }}\right|}^{2},$$

where σ denotes the total RCS, $\sigma_{k}$ the RCS of the $k^{\mathrm{th}}$ scatterer, and $d_{k}$ the distance between the $k^{\mathrm{th}}$ scatterer and the radar antenna. Therefore, the RCS of larger vehicles generally exceeds that of smaller vehicles, leading to more measured points scattered over a larger area. 

**Figure 13:**
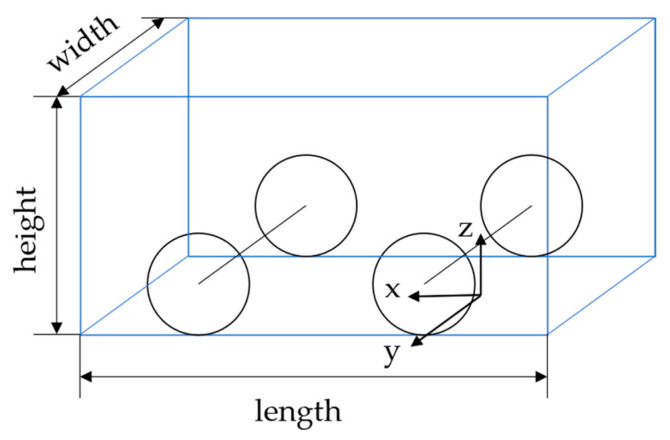
Simplified vehicle model with parameters length, width, and height.

**Figure 14:**
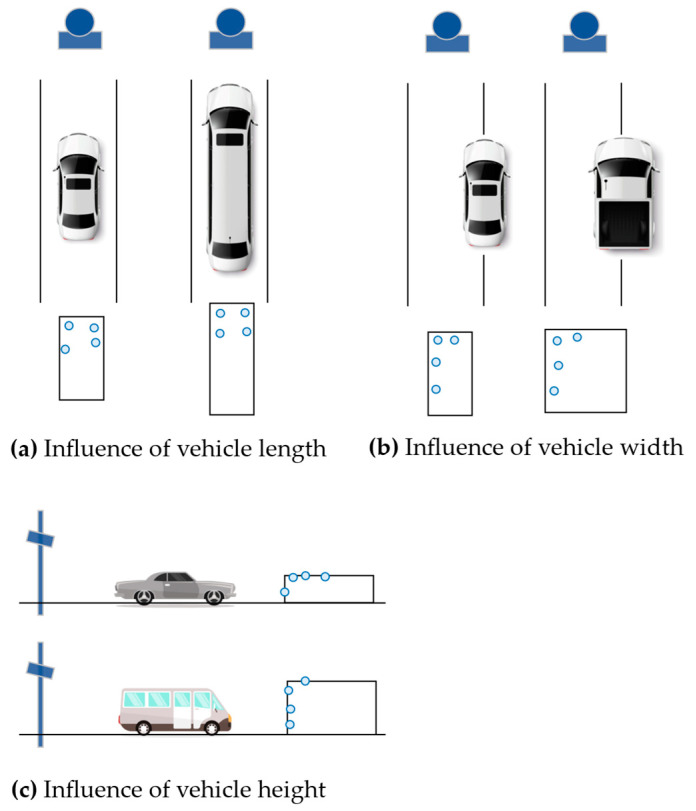
Variation in point cloud distribution regarding vehicle size.

Typically, we simplify the vehicle model as a rectangle with the parameters length, width, and height, as shown in Figure 13. Figure 14 depicts the influence of vehicle size on the point cloud distribution. As shown in Figure 14a, when the vehicle body partially enters or leaves the radar FOV as described in Section 3.2.3, the effective scatterer is located at the front and rear of the vehicle body. With a longer vehicle, a larger positioning error may be observed in the longitudinal direction. Similarly, Figure 14b shows that wider vehicles may generate larger lateral positioning errors. The vehicle height can block the radar beam from reaching the top surface, limiting the measured points to the vehicle’s rear, leading to longitudinal positioning error, as shown in Figure 14c. 

## 4. Simulation Results

As the ground truth of a target vehicle location can be difficult to obtain, we conducted simulated experiments to analyze the radar positioning accuracy based on radar installation height, vehicle location, posture, and size. Leveraging MATLAB’s Automated Driving Toolbox and Radar Toolbox, we created a roadside perception scenario and generated synthetic radar detections using a statistical radar model. The basic parameters of the simulated MMW radar are shown in Table 2. The installation position is defined by world coordinates (x, y, z). The installation pose is defined by angles in the order of (yaw, pitch, roll). The simulation is performed with a fixed step time of 0.01 s. 

**Figure 15:**
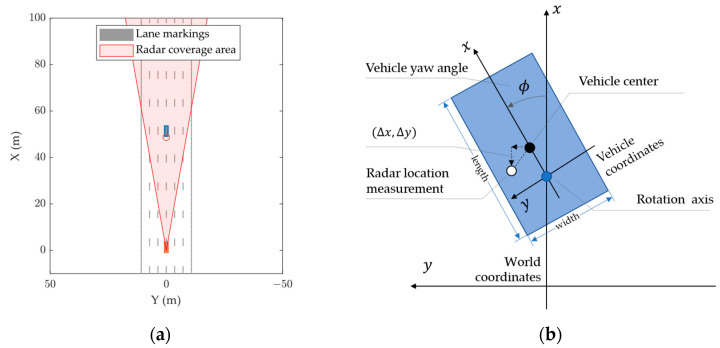
Simulation scenario setup: (**a**) radar FOV; (**b**) world and vehicle coordinate systems.

We set up a six-lane road section with a 5 m high gantry and installed the radar module at the center of the gantry, facing the x-direction with a 5° pitch angle downward. The radar FOV is shown in Figure 15a, where the orange rectangle represents the MMW radar, the blue rectangle the target vehicle, and the red conical area the radar FOV. The positioning error (Δx, Δy) is defined as the deviation in the radar location measurement from the vehicle center in the world coordinate system, as shown in Figure 15b. Only one vehicle is present at each experiment to avoid target occlusion. 

### 4.1. Influence of Radar Installation Height

**Figure 16:**
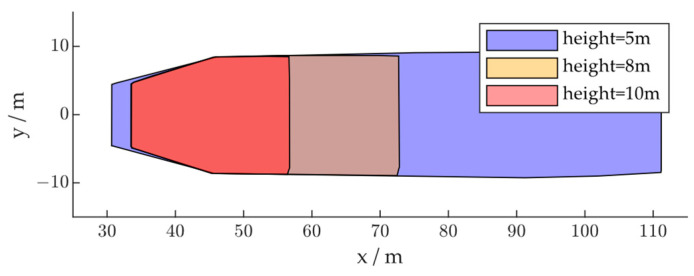
Radar FOV based on installation height.

To evaluate the influence of radar installation height, we set up the following radar installation scenario: (1) height: 5 m, pitch angle: 5°; (2) height: 8 m, pitch angle: 8.75°; (3) height: 10 m, pitch angle: 12.43°, so that the FOVs of different installation schemes all began at approximately the same position, as shown in Figure 16. The radar module was installed at the center of the road with zero roll angle and zero yaw angle. 

**Figure 17:**
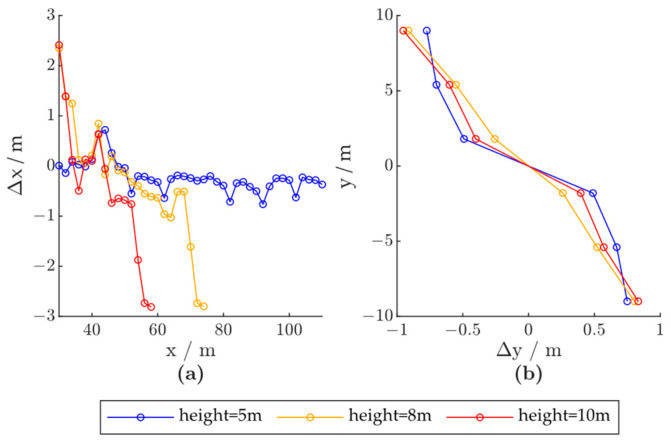
Radar positioning error based on radar installation height; x and y denote the vehicle positions in the world coordinate system, and Δx and Δy denote radar positioning errors in the x- and y-directions, respectively: (**a**) Δx - x; (**b**) y - Δy.

The simulation results are shown in Figure 16. As shown in Figure 17a, the x-direction positioning error characteristics under different installation heights are approximately the same, so that as the vehicle passes through, the positioning is first positively biased and then negatively biased. However, as the FOV decreases with the increase in installation height, the error characteristics are scaled in the x-direction, so that when the target vehicle is at the same location, the x-direction measurement from the radar installed at a higher location may deviate more from the ground truth. The error characteristics in the y-direction are not significantly influenced by the radar installation height, as shown in Figure 17b. Thus, although a higher installation of the radar module can lead to a reduced probability of target occlusion, the loss in FOV and positioning accuracy should also be considered in deploying roadside sensors. 

### 4.2. Influence of Radar Sampling Frequency

**Figure 18:**
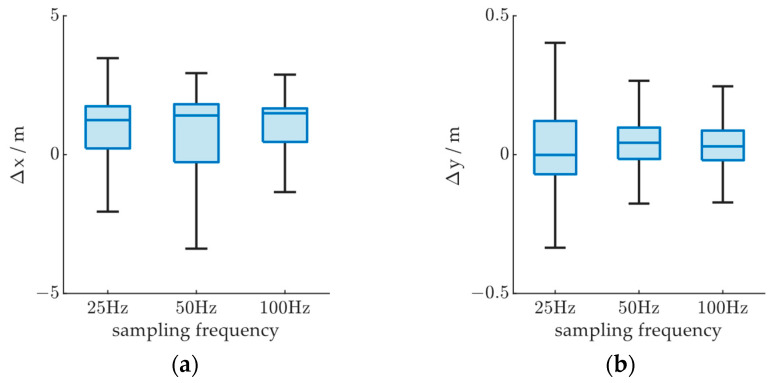
Radar positioning error based on MMW radar sampling frequency; Δx and Δy denote radar positioning errors in the x- and y-directions in world coordinates, respectively: (**a**) Δx based on sampling frequency; (**b**) Δy based on sampling frequency.

To evaluate the influence of radar sampling frequency, the target vehicle was set to run from (0,0) to (110,0) at a constant velocity of 20 m/s. A simple object tracking algorithm based on Kalman filter was used to acquire the vehicle trajectory. We observed the y-direction positioning error to evaluate the influence of the sampling frequency on the tracking algorithm and compared it against the x-direction positioning error characteristic. The sampling frequency was set to 25 Hz, 50 Hz, and 100 Hz as factors of the base simulation frequency. As shown in Figure 18a, the x-direction positioning error is not significantly influenced by the radar sampling frequency, while the y-direction positioning error is reduced as the sampling frequency increases, as shown in Figure 18b. This shows that a higher sampling frequency of the MMW radar can lead to more accurate tracking results, but the improvement is insignificant against the error caused by vehicle shape since the lateral positioning in the scenario is not influenced by the vehicle shape while the vehicle is set to run on the road center line, and the longitudinal positioning in the experiment is affected by the length and height, as will be shown in the following experiment. 

### 4.3. Influence of Vehicle Location

**Figure 19:**
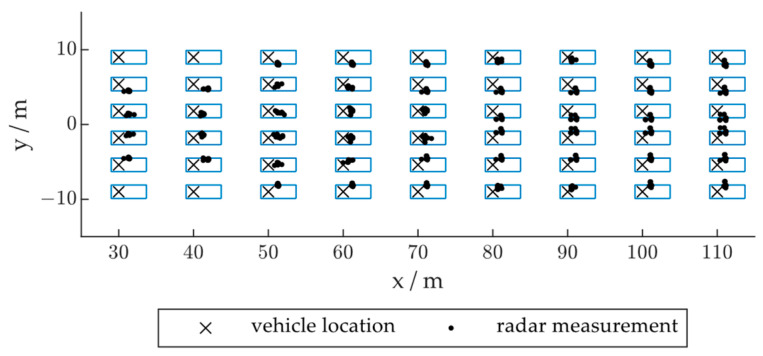
Distribution of the radar measurements regarding vehicle location.

**Figure 20:**
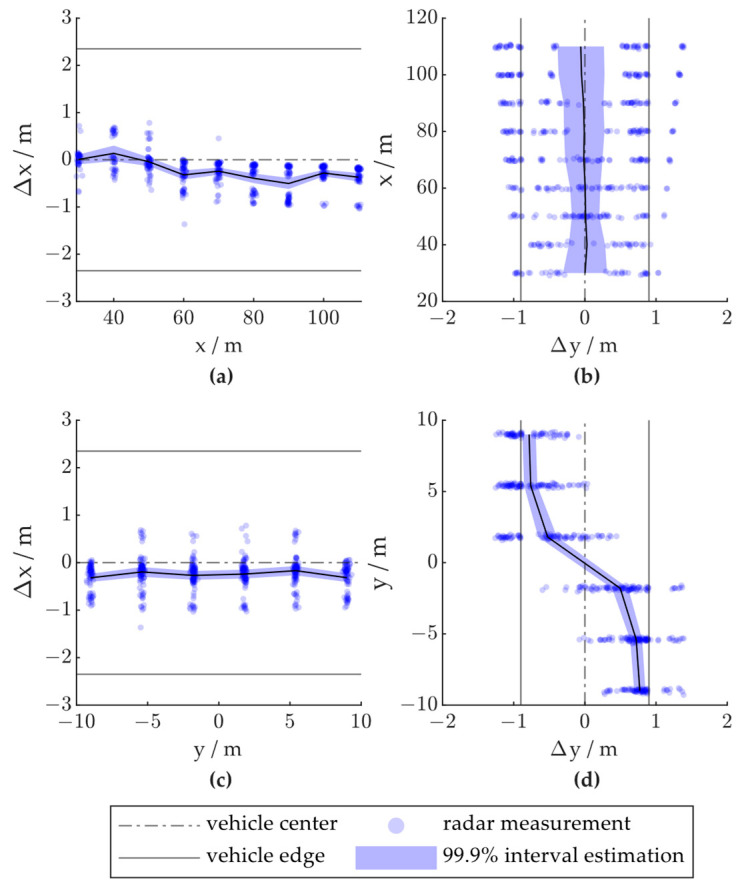
Radar positioning error based on vehicle location; x and y denote the vehicle positions in the world coordinate system, and Δx and Δy denote radar positioning errors in the x- and y-directions, respectively: (**a**) Δx - x; (**b**) x - Δy; (**c**) Δx - y; (**d**) y - Δy.

To analyze the influence of vehicle position, we divided the road into several sections. As shown in Figure 19, the distribution of the radar measurements varies considerably when the vehicle is located on different sections of the road. Figure 20 shows the distribution of the radar positioning error based on the vehicle location in the x- or y-direction. As shown in Figure 20a, as the vehicle passes through the radar FOV, the radar positioning in the x-direction is first slightly positively biased and then negatively biased. This is due to the distribution of the radar point cloud gradually moving backward over the vehicle body, as described in Section 3.2.3. Figure 20b shows that the y-direction deviation is larger when the vehicle has not fully entered the FOV or is driving away from the FOV. As shown in Figure 20c, the lateral position of the vehicle may not influence the longitudinal positioning accuracy, and when observed on the lane level, the radar positioning generally underestimates the vehicle distance in the x-direction. Figure 20d shows that when the vehicle is located on the right side of the radar central beam, the positioning results are biased to the left and vice versa. The height and width of the vehicle caused this phenomenon by blocking the radar signal from reaching the other side of the vehicle, resulting in side-only detections.

### 4.4. Influence of Vehicle Posture

**Figure 21:**
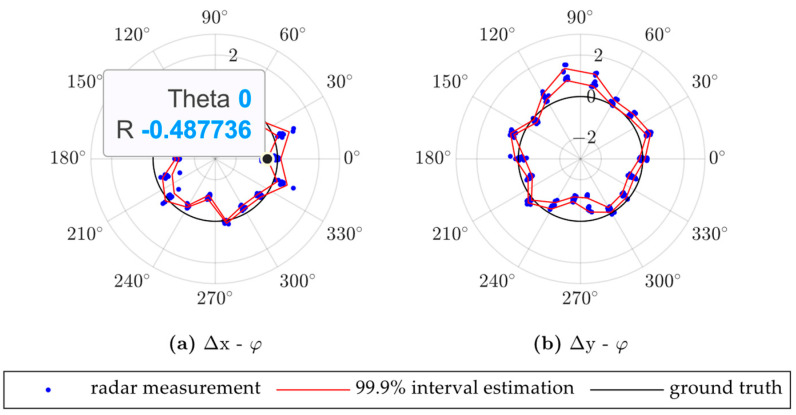
Radar positioning error based on vehicle yaw angle; Δx and Δy denote radar positioning errors in the x- and y-directions in world coordinates, respectively, and φ denotes the vehicle yaw angle.

To evaluate the influence of the vehicle posture, we fixed the location of the vehicle to (50,0), that is, 50 m from the radar installation position on the road center line, and rotated the vehicle around the center of the rear axle, as shown in Figure 16b. The results are shown in Figure 21. Figure 21a shows that fewer range estimations may appear when the yaw angle approaches ±90° and −180°. Figure 21b shows that as the vehicle center shifts to the right or left while the vehicle rotates, the radar positioning may drift in the opposite direction, as described in Figure 20d. Thus, a larger positioning error may appear when the vehicle engages in lane changing or other behaviors that may cause large variations in its yaw angle. Conversely, the radar positioning is more accurate when the vehicle runs in a straight line. 

### 4.5. Influence of Vehicle Size

**Figure 22:**
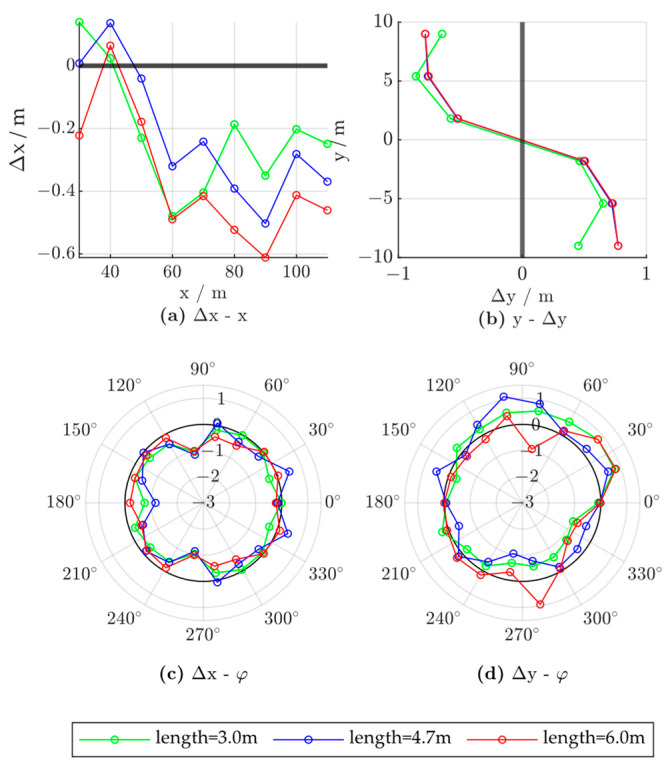
Radar positioning error based on vehicle length; x and y denote the vehicle positions in the world coordinate system, Δx and Δy denote radar positioning errors in the x- and y-directions, respectively, and φ denotes the vehicle yaw angle.

**Figure 23:**
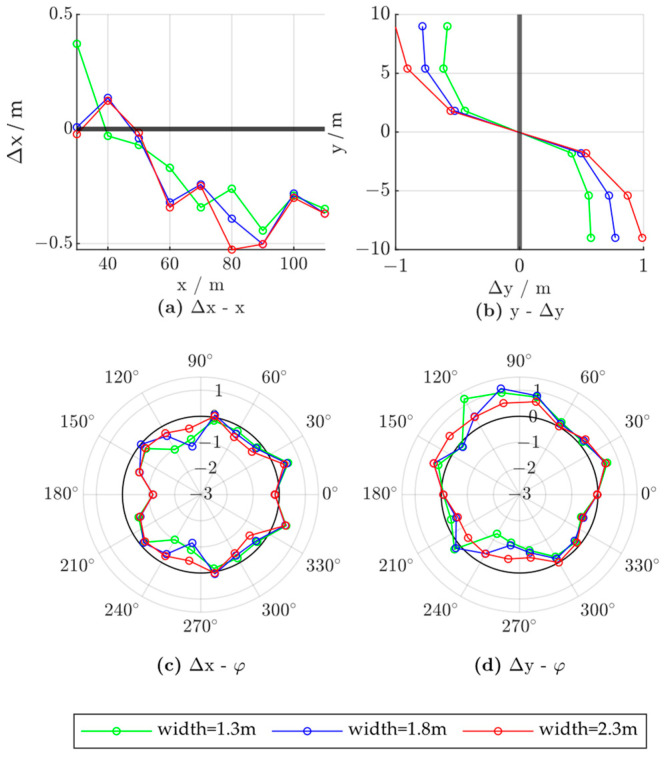
Radar positioning error based on vehicle width; x and y denote the vehicle positions in the world coordinate system, Δx and Δy denote radar positioning errors in the x- and y-directions, respectively, and φ denotes the vehicle yaw angle.

**Figure 24:**
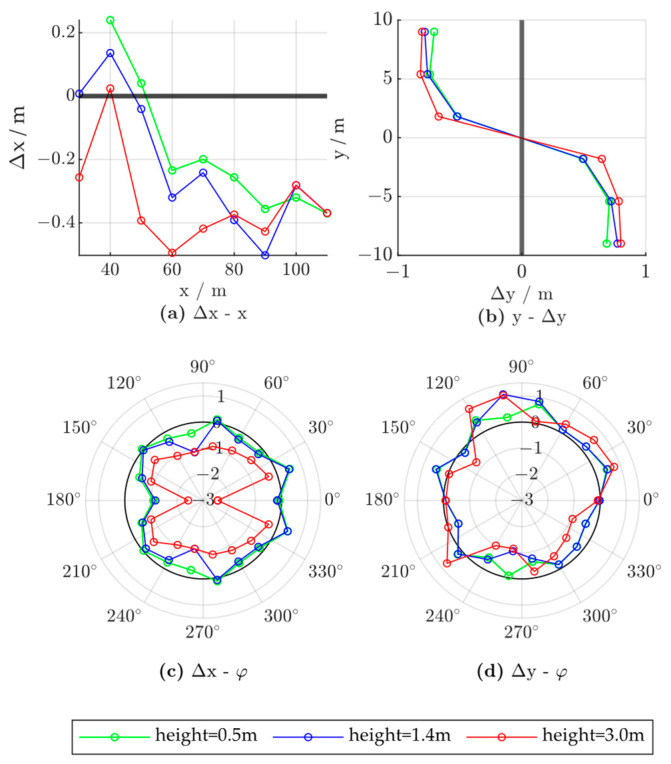
Radar positioning error based on vehicle height; x and y denote the vehicle positions in the world coordinate system, Δx and Δy denote radar positioning errors in the x- and y-directions, respectively, and φ denotes the vehicle yaw angle.

To evaluate the influence of the vehicle size, we compared the error characteristics of vehicles with different lengths, widths, and heights. We fixed the vehicle location to (50,0) and set the vehicle yaw angle to 0. The control group vehicle was of size 1.8 × 4.7 × 1.4; that is, 1.8 m in width, 4.7 m in length, and 1.4 m in height. Figure 22, Figure 23 and Figure 24 show the experimental results, where the red lines represent the characteristics of the larger vehicle (in length, width, or height), the blue lines represent the vehicle of controlled size, and the green lines represent the smaller vehicle. 

The influence of vehicle length was evaluated at 3.0, 4.7, and 6.0 m. As shown in Figure 22a, the x-direction deviation due to the vehicle position increases with the vehicle length. Thus, the roadside detection results should be corrected with the target vehicle parameters to achieve greater positioning accuracy. In this case, the radar x-direction measurement should be adjusted with the vehicle length according to the error characteristics presented in Figure 22a. The lateral positioning error characteristics based on the lateral vehicle location and the longitudinal positioning error characteristics based on the vehicle yaw angle are not significantly influenced by the change in vehicle length, as shown in Figure 22b,c. A larger lateral positioning error at a ±90° yaw angle is observed for a longer vehicle, as shown in Figure 22d. 

The influence of vehicle width was evaluated at 1.3, 1.8, and 2.3 m. As shown in Figure 23a,d, the y-direction positioning error due to the influence of the vehicle position and yaw angle is larger, while the x-direction error characteristics broadly remain constant, as shown in Figure 23a,c. Thus, the lateral positioning of the target should be adjusted based on its width information, and greater adjustments should be made for wider vehicles, such as trucks and buses. 

The influence of vehicle height was evaluated at 0.5, 1.4, and 3.0 m. As shown in Figure 24a, radar may underestimate the target distance in the longitudinal direction when the vehicle is close to the radar module, as taller vehicles can block the radar beam from reaching the front part of the vehicle. Therefore, when the radar targets include vehicles of significantly different heights, such as double-decker buses or cargo trucks, the longitudinal positioning should also be adjusted based on the vehicle height. Moreover, when the vehicle is taller, reduced overall longitudinal positioning based on the vehicle yaw angle is observed, as shown in Figure 24c. The lateral positioning error characteristics are not significantly influenced by vehicle height, as shown in Figure 24b,d. 

## 5. Guidelines for MMW Radar Data Processing

Based on the experiment results, we propose the following guidelines for MMW radar data processing in risk assessment and early warning systems. 

### 5.1. Data Filtering Based on Vehicle Location

**Figure 25:**
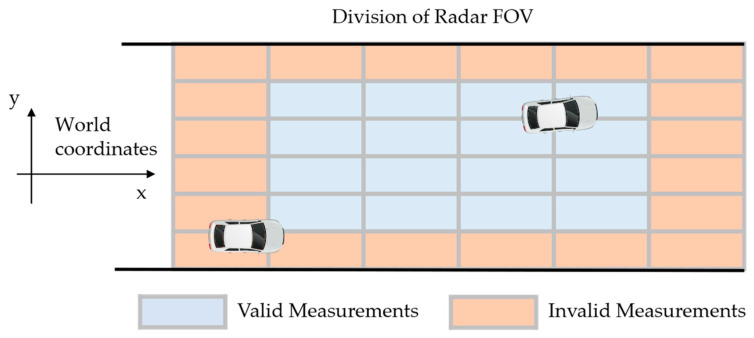
Data filtering based on vehicle location.

As shown in the previous results, the positioning error is greater when the vehicle has not fully entered the FOV or is driving away from the FOV. Therefore, the original FOV can be divided into several zones, as shown in Figure 25. Position measurements located in zones found with a large positioning error can be filtered out to eliminate unnecessary calculations and interference to other data sources, but zone occupancy must be retained for safety concerns. For the calculation of the risk indicators, the worst possible scenario within the zone must be assumed. 

### 5.2. Data Filtering Based on Vehicle Posture

**Figure 26:**
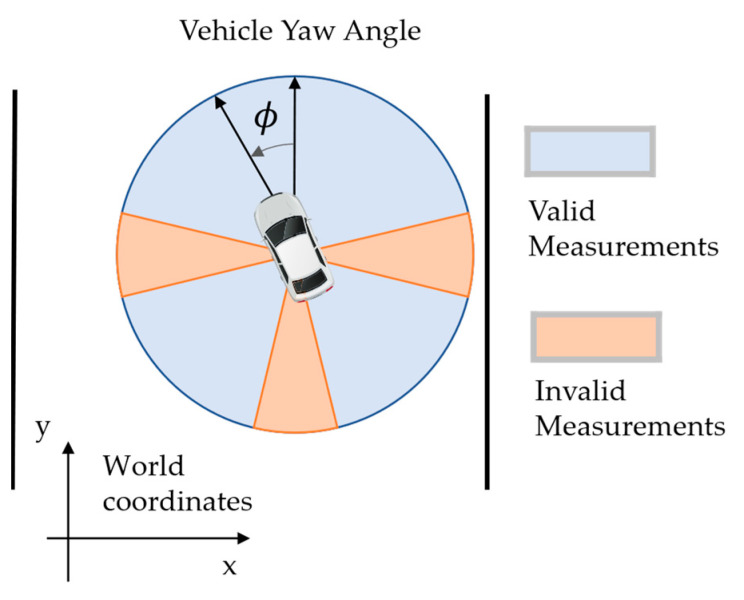
Data filtering based on vehicle posture.

Similarly, greater positioning error is found when the vehicle yaw angle is near ±90° and 180°. Therefore, a partitioning on the vehicle yaw angle can be implemented to filter out unreliable position measurements, as shown in Figure 26. 

### 5.3. Measurement Adjustment Based on Vehicle Size

As shown in Figure 22, Figure 23 and Figure 24, radar positioning accuracy is significantly influenced by the size of the vehicle. For longitudinal position measurement, when the vehicle is closer to the radar module, the measurement should be compensated with vehicle height. A positive adjustment should be made for vehicles with greater height, while a negative adjustment should be made for vehicles with lesser height. When the vehicle is farther away, a positive adjustment in proportion to the vehicle length should be made for longitudinal position measurement. For lateral position measurement, a negative adjustment in proportion to the vehicle width should be made. 

## 6. Conclusions

In this paper, we analyze the influence of radar installation height, vehicle location, posture, and size on the positioning accuracy of MMW radar. In the numerical simulation, we set up a roadside perception scenario where the MMW radar is mounted at the center of a gantry, overlooking the road traffic. The following conclusions are drawn from the experiment results.

- Influence of radar installation height: When the radar is installed at a higher position with a greater pitch angle to monitor the same section of road, a larger longitudinal positioning error is observed when the vehicle is driving away from the radar FOV.
- Influence of radar sampling frequency: Greater tracking error on the y-direction is observed when the sampling frequency is lower. The tracking error on the x-direction is not significantly influenced by the sampling frequency.
- Influence of vehicle location: When the vehicle passes through the radar FOV, the radar positioning in the longitudinal direction is first positively and then negatively biased. In the lateral positioning, the radar positioning biases to the left when the vehicle locates on the right side of the radar central beam, and vice versa.
- Influence of vehicle posture: A large positioning deviation is observed when the vehicle yaw angle is at ±90°.
- Influence of vehicle size: When the vehicle is closer to the radar module, the vehicle height can severely affect longitudinal positioning. The vehicle length causes longitudinal positioning errors when the vehicle is further from the radar module. A greater lateral positioning error is observed when the vehicle is wider.

Based on the above conclusions, a general guideline for MMW radar data processing in risk assessment and early warning systems is proposed to acquire more accurate risk information. In the future, real-world experiments can be conducted to verify the simulation results and the effectiveness of the proposed guidelines. Plenty of work still needs to be carried out to tackle the high environment noises, target occlusions, and efficient sensor calibration. 

## Figures and Tables

**Table 1:** Effective scatterers and the number of measured points in different longitudinal locations.

Vehicle Longitudinal Location	Effective Scatterer	Measured Points
Partial entry	Front	Few
Full entry	Body and rear	Many
Driving away	Rear	Some

**Table 2:** Parameters of the simulated MMW radar.

Parameters	Value	Unit
Center frequency	77 × 10^9^	Hz
Range limits	150	m
Azimuthal field of view	20	degree
Elevation field of view	5	degree
Range rate limits	100	m/s
Azimuth resolution	4	degree
Range resolution	2.5	m
Range rate resolution	0.5	m/s
Update rate	100	Hz
Installation position	(0, 0, 5)	m
Installation pose	(0, 5, 0)	degree

## Data Availability

Not applicable.
